# Diagnosis and Prognostic Implications of Primary Intraosseous Carcinoma: A Case Report and Literature Review

**DOI:** 10.7759/cureus.69155

**Published:** 2024-09-11

**Authors:** Nicholas A McDonald, William Montagne, Sonal Shah, Joshua J Goldman, Jo-Lawrence Bigcas

**Affiliations:** 1 Otolaryngology, Washington State University, Spokane, USA; 2 Otolaryngology-Head and Neck Surgery, University of Nevada Las Vegas School of Medicine, Las Vegas, USA; 3 Oral and Maxillofacial Surgery, University of Nevada Las Vegas School of Medicine, Las Vegas, USA; 4 Plastic Surgery, Vegas Plastic Surgery Institute, Las Vegas, USA

**Keywords:** carcinoma of unknown primary (cup), mandibular carcinoma, primary intraosseous carcinoma, squamous cell carcinoma of unknown primary, squamous cell carcinoma (scc)

## Abstract

Primary Intraosseous Carcinoma (PIOC) is a rare and aggressive squamous cell carcinoma (SCC) derived from remnants of odontogenic epithelium with no initial connection to oral mucosa. Due to the rarity of the disease, etiology and epidemiology are not clearly defined. The most affected site is the posterior mandible, and clinical features include swelling of the jaw, jaw pain, and sensory disturbances. Given the similarities of PIOC to other odontogenic carcinomas, diagnosis is often difficult, resulting in delays in intervention. Treatment of PIOC of the mandible includes surgery alone, surgery with adjuvant radiotherapy or chemotherapy, and free flap reconstruction. PIOC prognosis is poor, with the lymph nodal status acting as an important indicator. We present a case of a 60-year-old female who presented with a left submandibular mass initially thought to be SCC of unknown primary origin. Further investigation led to a final diagnosis of PIOC of the mandible. Clinical, radiological, and histological features of PIOC will be discussed.

## Introduction

Primary intraosseous carcinoma (PIOC) is a rare neoplasm, defined as a squamous cell carcinoma of the jaw developing from remnants of odontogenic epithelium or an odontogenic cyst with no initial connection to oral mucosa [1-9]. Described by Loos as a central epidermoid carcinoma of the jaw in 1913, Pindborg approved the term primary intraosseous odontogenic carcinoma in 1972 [5,10,11]. In 2005, WHO classified PIOC into three subtypes according to histogenesis: a solid-type carcinoma, carcinoma arising from the odontogenic cyst, and carcinoma arising from a keratocystic odontogenic tumor [2,7,9-11]. Additionally, the classification of PIOC of the jaw based on possible origin has been established. 

Primary intraosseous neoplasms account for 12% of all cases of oral cancer and arise primarily from odontogenic cysts that include keratocystic odontogenic tumors and dentigerous cysts. Rarely the neoplasm originates from residual periapical cysts [2]. Definitive diagnosis of PIOC is often difficult, and diagnostic criteria remain elusive as the lesion must be differentiated from other odontogenic carcinomas such as malignant ameloblastoma, SCC arising from overlying oral mucosa, primary tumors of the maxillary sinus or nasal mucosa, and from tumors that have metastasized to the jaw from other primary sites [7]. 

Most common in the fifth to seventh decade of life, PIOC presents with painful swelling in the jaw region; however, some patients present as asymptomatic, with identification of the tumor occurring incidentally through a routine radiograph [5]. Common radiographic features include an osteolytic appearance with ill-defined irregular margins [2]. Although an approved protocol has yet to be established, the primary treatment for PIOC is wide local resection, with enbloc excision or radical resection of the affected bone advised [11]. With prognostic factors that include lymph nodal involvement and histological grading, prior studies have reported five-year survival rates of 30-40% with local recurrence of 50-60% [11]. Given the prevalence of metastasis to cervical lymph nodes, other modalities such as radiation and chemotherapy may also be performed as adjunctive treatments [1,5,6,11]. 

Here, we present an interesting case of a 60-year-old female with PIOC of the mandible arising de novo, initially diagnosed as squamous cell carcinoma of unknown primary origin. Upon proper diagnosis of the neoplasm, left segmental mandibulectomy, osteo-septo-cutaneous free fibular flap, and mental nerve reconstruction were carried out following accurate staging. Postoperatively, adjuvant radiotherapy was performed due to local recurrence rates. Through a review of the clinical, radiological, and microscopic characteristics of PIOC, this case will add to the limited literature available and will help better characterize this entity. This case was presented at the American Head & Neck Society Conference on July 8th, 2023, in Montreal, Canada. 

## Case presentation

A 60-year-old female with a past medical history of hypertension, hyperlipidemia, and smoking, presented with a one-year history of a slow-growing, painless left submandibular mass. The patient reported a 10-year history of smoking and a long-standing history of weekly social drinking. Aside from the mass, she reported no other symptoms. 

**Figure 1:**
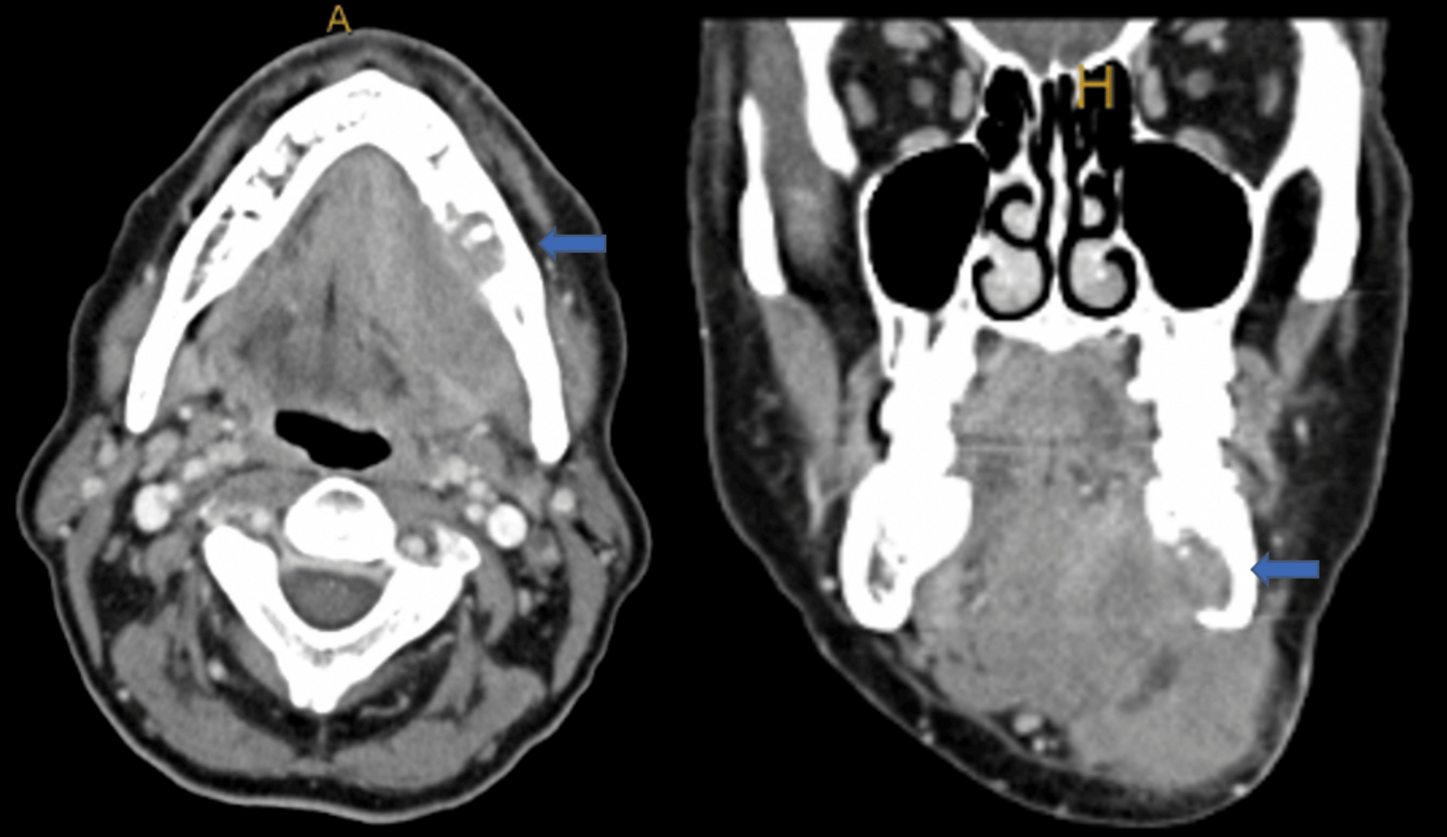
Pre-operative CT Pretreatment CT neck pre-op shows soft tissue mass breaking through the lingual cortex of the mandible. The blue arrow points to a mass involving the left mandibular cortex.

Upon physical examination, she demonstrated a firm 3 cm x 2 cm left submandibular triangular mass at the level of the inferior border of the mandible. No other palpable cervical adenopathy was identified. Transoral examination demonstrated healthy and intact teeth without any evidence of periodontal breakdown or a mucosal primary tumor. Flexible fiberoptic examination demonstrated no sino-nasal, pharyngeal, or laryngeal mucosal findings. Additionally, no alteration or ulceration of the overlying mucosa was present. Fine needle aspiration of her tumor was consistent with squamous cell carcinoma. Head and neck radiographic imaging included computed tomography scan (CT), Magnetic resonance imaging (MRI), and positron emission tomography (PET)/CT to determine the primary site of metastasis and the extent of the lesion. A review of the CT and MRI suggested inferior lingual cortical erosion of the mandible with the submandibular mass emanating from the soft tissue in the marrow space, with possible left neck lymphadenopathy (Figure 1). PET/CT scan demonstrated no distant metastases.

**Figure 2:**
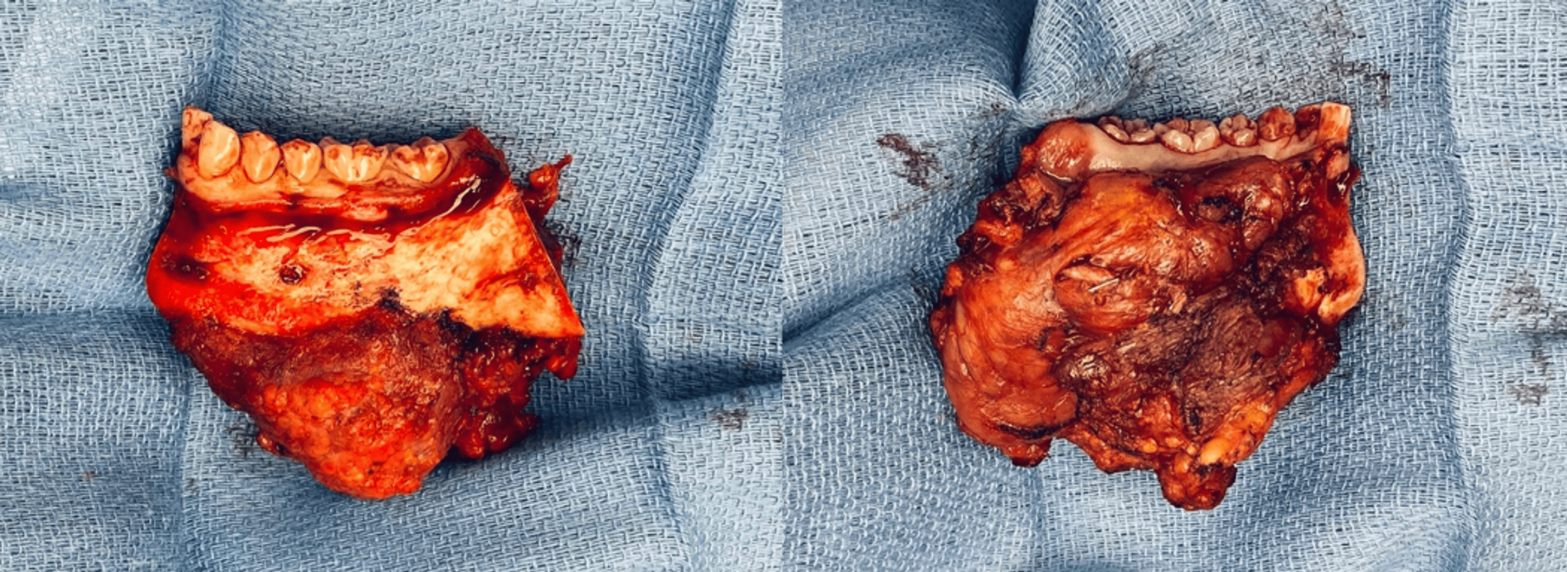
Intra-operative left mandible composite resection of primary intra-osseous carcinoma The lateral aspect (left) and medial aspect (right) of the left mandible.

The patient was subsequently referred to the multidisciplinary team, where she was indicated for surgical management, followed by adjuvant radiation +/- chemotherapy, pending pathology results. The patient underwent oral cavity composite resection, left modified radical neck dissection, and fibular free flap with microvascular anastomosis. During the resection, a margin of gingivoalveolar mucosa was resected (Figure 2). A frozen section on the mucosal margins confirmed no mucosal tumor. The patient’s post-operative hospital course was uncomplicated. 

**Figure 3:**
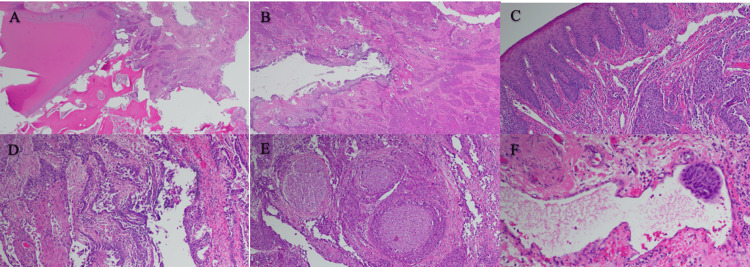
Pathology slides of PIOC A) Showing tumor at 2x low power adjacent to the tooth root. B) 4x power showing cyst and tumor islands. C) 10x power showing normal surface next to tumor island D) 10x power showing tumor coming from the cyst E) 10x power showing perineural invasion of the tumor. F) 20x power showing intravascular tumor infiltration.

A final pathology report demonstrated no tumor at the mucosal and bony margins of the specimen. There was no evidence of continuity with the surface mucosa. Areas of odontogenic cyst showing dysplastic transformation of the lining with islands of neoplastic epithelial cells branching from this cyst lining were identified (Figure 3). These microscopic features led to a final diagnosis of primary intraosseous carcinoma. Multiple lymph nodes were found to be involved, as well as perineural and lymphovascular invasion. Pathologic TNM staging was pT4aN2bM0. The patient was administered adjuvant radiation alone without chemotherapy. Follow-up at three years shows no clinical or radiographic disease.

## Discussion

Primary intraosseous carcinoma (PIOC) is a rare neoplasm described as a squamous cell carcinoma that arises from residues of odontogenic epithelium entrapped within the jaw, with no connection to oral mucous membranes [1-5,8,9,11]. From the first description by Loos in 1913 of an intra-alveolar carcinoma of the jaw, alterations to the term and defining characteristics of PIOC have been identified. The classification of PIOC of the jaw has been established and modified to account for various possible origins (Table 1). PIOC is the most common malignant tumor of the jaw; however, due to its rarity and poor prognosis, incidence, prevalence, and etiology are still unknown [2,3,12]. 

**Table 1:** Classification of primary intraosseous carcinoma of the jaw based on possible origin PIOC: Primary intraosseous carcinoma

Type 1	PIOC such as odontogenic cysts
Type 2A	Malignant ameloblastoma
Type 2B	Ameloblastic carcinoma arising de novo or ex odontogenic cyst
Type 3	PIOC arising de novo: keratinizing type & Nonkeratinizing Type
Type 4	Intraosseous mucoepidermoid carcinoma (IMEC)

Epidemiologically, PIOC occurs primarily in adults, with a predilection for males with a male-to-female ratio of 2.5:1 [2]. Incidence increases with age, with risk factors that include a history of smoking and drinking, and co-morbidities like hypertension and diabetes [13]. The peak incidence of PIOC is in the fifth to seventh decade of life, with a mean age of 54 years at the time of diagnosis. A comprehensive analysis of epidemiological and other associated PIOC characteristics in past literature shows similar trends (Table 2). In a study by Zwetyenga et al., the age range was 4-76 years old [5]. Similarly, in a pooled analysis of world literature by Thomas et al., 35 cases of PIOC were reviewed with a mean age of 52.3 years and an age range of 4-81 years old [8]. Most patients within the pooled analysis were over the age of 65 years (38.7%), and the posterior mandible was the most common tumor site, although cases with anterior mandible and maxillary involvement have been reported [1,6-8,13,14]. In studies conducted by Bodner et al. [9] and Huang et al. [7], 79% and 84.6% of tumors occurred in the posterior mandible, respectively, aligning with the tumor site seen in our patient. Additionally, Wang et al. examined the location site of 80 patients diagnosed with PIOC, with 51.2% of tumors identified on the mandible ramus, 31.2% identified on the mandible body, and 17.6% of tumors located on the maxilla [13]. Tumors are primarily unilateral, with 95% of patients presenting with unilateral lesions in Wang et al. study [13]. 

**Table 2:** Epidemiological factors, nodal status, treatment, and reconstruction modalities in past literature and our case n., number; N, lymph node metastasis; P.M – Posterior Mandible; n.a. Not Available; S – Surgery; R – Radiotherapy; C – Chemotherapy; OS – Overall Survival; *Study includes all types of primary jaw tumors without specification for primary intraosseous carcinoma **2 Patients lost to follow-up; 37 cases analyzed for treatment; ***PIOC type III selected for study.

Author/Date	n. of cases (Total)	Epidemiological Factors	Tumor Site	%	N status	%	Treatment	Recurrence and OS
Present Case	1	Age – 60 Female	P.M		+		S + R	
Wang et al. * 2021 [13]	80	Age range – 11-92 Mean age – 51.5	Mandible – 66 Maxilla – 14	82.5 17.5	N (-) – 64 N (+) – 16	80 20	n.a	DFS – 60.6% OS – 73.2%
Morais et al. 2021 [15]	257	Age range – 5-89 Mean age – 57.3	Mandible – 225 Maxilla – 32	87.5 12.4	N (-) – 78 N (+) – 33	30.4 12.8	S - 102 S + R – 89 S + C – n.a. S + R + C - 20	2-Year OS – n.a. 5-Year OS – 44.6%
Wenguang et al. 2016 [4]	77	Age range – 37-81 Mean age – 58.8	Mandible – 55 Maxilla – 22	71.4 28.6	N (-) – 57 N (+) – 20	74 26	S - 43 S + R – 19 S + C – 6 S + R + C – 9	2-Year OS – 68.9% 5-Year OS – 38.8%
Bodner et al. 2011 [9]	116	Age range – 1.3-90 Mean age – 60.2	Mandible – 92 Maxilla – 24	79 21	N (-) – n.a N (+) – 6		S - 53 S + R – 44 S + C – 7 S + R + C – 7	2-Year OS – 62% 5-Year OS – 38%
Huang et al.** [7] 2009	39	Age range – 24-82 Mean age – 54	Mandible – 37 P.M – 33 Maxilla – 2	94.9 89.2 5.1	N (-) – 18 N (+) – 17	48.6 45.9	S - 14 S + R – 18 S + C – 0 S + R + C – 5	2-Year OS – 69.8% 5-Year OS – 36.3%
Thomas et al.*** [8] 2001	35	Age range – 4-81 Mean age – 52.3	Mandible – 30 P.M - 27 Maxilla - 4	85.7 77.1 11.4	N (-) – 24 N (+) – 11	68.4 31.6	S - 14 S + R – 8 S + C – 1 S + R + C – 2	2-Year OS – 62.1% 5-Year OS – 37.8%
Zwetyenga et al. [5] 2001	36	Age range – 4-76 Mean age – 54	Mandible – 33 Maxilla – 3	92 8	N (-) – 26 N (+) – 10	72.2 27.8	S – 16 S + R – 13 S + C – 2 S + R + C – 2	2-Year OS – 60.5% 4-Year OS – 39.9%

Despite the pathogenesis of PIOC remaining unclear, reports have suggested that long-standing chronic inflammation may be the primary predisposing factor for malignant transformation in cyst epithelium. According to Bodner et al., the mechanism of inflammation-induced carcinogenesis involves the formation of reactive oxygen metabolites, causing damage to cell membranes, proteins, and DNA [9]. This damage leads to a compensatory proliferative process of neoplastic cells against the normal physiological apoptotic mechanism, consequently resulting in uncontrolled cellular proliferation [9]. Considering the suspected role of chronic inflammation, alcohol and tobacco use have been examined as the risk factors due to increased cellular oxidative stress from these substances. However, a clear connection between alcohol and tobacco use and PIOC pathogenesis has yet to be established. 

The clinical presentation of PIOC is non-specific and typically includes varying symptoms depending on tumor size, location, and type. Patients commonly present with jaw pain and swelling, erosion of buccal and lingual cortical plates, and chronic sinus tract findings [1-3,10,11,13]. Additional findings of paresthesia or numbness of the mandibular nerve are frequent [7]. Alternatively, like our patient, asymptomatic cases have been reported in past literature, typically present at early stages of PIOC onset, which may delay diagnosis and treatment [5,9,11]. Owing to an asymptomatic development of PIOC, lymph node invasion may be present when a biopsy is performed, indicating a worse prognosis for the patient. In the Bodner et al. study, swelling and pain of the jaw were the most common clinical features at 32% and 24%, respectively, with 11% of patients being asymptomatic [9]. Similar trends were seen in Thomas et al. pooled analysis of 33 cases of PIOC, where 54.8% of patients presented with jaw pain, 51.7% with swelling of the jaw, and 16.1% presented with sensory disturbances [8].

A radiographic examination can assist with the detection of PIOC, with most cases exhibiting radiolucency with a wide variation in size, shape, and border appearance [6,11]. Kaffe et al. examined 24 cases of radiologic features of PIOC, with 87% of cases being radiolucent and 13% of cases classified as mixed radiolucent-radiopaque [3,14]. In addition, borders of lesions were diffuse in 45% of cases and defined but non-corticated in 57%, with non-corticated borders indicating aggressive development of the tumor [10,14]. Suei et al. suggested that slow-growing PIOCs had well-defined, smoothly contoured borders, while PIOCs that grew rapidly had poorly defined, ragged borders [6]. The variation in size and shape makes PIOCs difficult to distinguish from other benign or malignant tumors within the jaw region, emphasizing the importance of PIOC being considered in the differential diagnosis of radiolucent lesions of the jaw [14,15]. In our case, the lesion extended through the posterior submandibular cortex with a well-defined corticated border (Figure 1). 

Microscopically, PIOC typically presents as well-defined or moderately well-defined SCCs that may be keratinized or non-keratinized [12]. PIOC exhibits sheets or islands of malignant epithelial cells, with abundant keratin formation scattered over densely fibrous stroma and the absence of a clear cell component [1,3,11]. Additionally, PIOC reveals a distinct odontogenic pattern with basal-type cells forming alveoli or arranged in a plexiform pattern with palisading of peripheral cells, with nuclei oriented away from the basement membrane (2,3,5,7]. Zwetyenga et al. reported that the tumor is composed of sheets, islands, and strands of squamous cells with cellular pleomorphism, nuclear hyperchromatism, and mitotic activity, with some cases having distinct odontogenic patterns [5]. Our case fulfilled histologic criteria determined by Suei et al., with keratinizing islands of malignant epithelial cells derived from odontogenic epithelium [6]. However, historically, histologic features of PIOC are often difficult to distinguish from any other squamous cell carcinoma of squamous epithelium (i.e., oral mucosa), thus histologic examination is not a definitive diagnostic factor [7,13]. 

In our case, considering non-specific clinical features, radiographic features that met the criteria for PIOC, and histopathological findings of keratinized formation and tumor islands with intravascular tumor infiltration, PIOC was the definitive diagnosis (Table 3). 

**Table 3:** Diagnostic criteria according to Suei et al. with comparison to the case presented in this report Suei et al. [6]

Diagnostic criteria	Patient case
Absence of another primary tumor on chest radiograph	CT and PET scans showed cortical erosion of the mandible with no other lesions identified
Absence of an ulcer in oral mucosa overlying the tumor except in cases of other factors	Histopathological review indicated no continuity of overlying surface mucosa
Histological evidence of epithelial lining transition into squamous cell carcinoma infiltration	Histology indicated cyst and keratinizing tumor islands with intravascular tumor

Due to the highly malignant nature of PIOC, management should be aggressive. Treatment is principally wide local resection, with enbloc excision or radical resection advised [1,10,11]. The treatment adopted for our patient was a left segmental mandibulectomy, tracheostomy, and ipsilateral neck dissection. Due to the presence of lymphadenopathies in radiological findings, this aggressive approach was taken due to the extent and localization of the lesion within the posterior mandible. In the present case, simultaneous reconstruction was necessary, thus the vascularized osteo-septo-cutaneous fibular free flap is a reliable option for mandibular reconstruction to maintain functionality and aesthetical profile [10,11,13]. Adjunctive treatment modalities such as radiation and chemotherapy may also be performed in cases of PIOC with metastasis to cervical lymph nodes, along with surgical resection [4,7-9,11]. Our patient’s TNM stage of pT4N2bM0 warranted the use of adjunctive treatment through post-operative radiation. Huang et al. mentioned that patients who underwent surgery alone had better outcomes than patients who received adjuvant radiotherapy or chemoradiotherapy. However, for patients with advanced or high-grade tumors with a high likelihood of metastases, the use of postoperative adjuvant therapy is considered due to recurrence rates [7]. 

The prognosis for PIOC de novo is generally poor, with overall survival (OS) at two and four years of 60.5% and 39.9%, respectively [5]. Patients who underwent surgery and post-operative radiotherapy had a two-year survival probability of 61.3% [5]. In the Zwetyenga et al.'s study of 34 patients, 56% had recurrence. Prior studies have reported a five-year survival rate of 30-40% with a local recurrence of 50-60% [7,13]. In Wang et al.'s study, disease-free survival (DFS) was determined from the time of surgery to any recurrence or distant metastasis. Overall, DFS with follow-ups for 71 patients was 60.6%, with an OS rate of 73.2% [13]. A significantly poor prognosis was associated with factors such as smoking history, drinking history, and N+ status [13]. Due to our patient’s successful outcome at a two-year follow-up with advanced age and a 10-pack-year smoking history, we postulate that surgical intervention and reconstruction were successful. 

Metastasis to regional lymph nodes is a poor prognostic indicator for patients with PIOC, and previous literature has noted metastasis to cervical lymph nodes being observed in 50% of all cases [4,11,12]. A systematic review of 257 cases of PIOC of the jaw by de Morais et al. showed lymph node metastasis in 13.8% of patients living and 35% of patients that were examined after death; however, their analysis reported lymph node metastasis as a statistically insignificant association with survival rates [15]. In Thomas et al. pooled analysis, 31.4% of cases showed metastasis to regional lymph nodes at the time of presentation [8]. Due to the presence of left neck lymphadenopathy on the CT neck and PET scan in our patient, ipsilateral neck dissection was performed. This also prompted us to indicate adjunctive radiation therapy treatment due to nodal status.

## Conclusions

Given the rarity of PIOC, the literature is not extensive, and the etiology of the disease is still unclear. Delay in treatment is common due to non-specific symptoms and diagnostic similarities to other benign and malignant neoplasms. Once PIOC develops metastasis, accompanied by other factors such as smoking history and drinking history, outcomes are generally poor. This case presents an opportunity for clinicians to expedite diagnosis considering the historical recurrence of delayed detection due to unknown etiology and similar features to other benign lesions. Further research into etiological factors and defining characteristics of PIOC de novo may elucidate the underlying pathogenesis of the disease to allow for earlier detection and prevention. Clinicians should always consider the possibility of PIOC for radiolucent lesions of the posterior mandible with asymptomatic presentation.
